# Spatial Heterogeneity of Phytoplankton Taxa and Functional Groups Under Multidimensional Environmental Factors in Karst Urban Rivers

**DOI:** 10.3390/biology15120981

**Published:** 2026-06-22

**Authors:** Ting Wu, Qiuhua Li, Heng Wang, Yan Chen, Lan Chen, Qian Chen, Yongxia Liu

**Affiliations:** 1 Key Laboratory for Information System of Mountainous Area and Protection of Ecological Environment of Guizhou Province, Guizhou Normal University, Guiyang 550001, China; wuting98828@163.com (T.W.); gznuchenqian@126.com (Q.C.); 2 Guizhou Key Laboratory of Advanced Computing, Guizhou Normal University, Guiyang 550001, China; 100396569@gznu.edu.cn (H.W.); cy@gznu.edu.cn (Y.C.); 3 Guizhou International Science & Technology Cooperative Base of Aquatic Ecology Research, Guiyang 550001, China; 4 School of Cyber Science and Technology, Guizhou Normal University, Guiyang 550001, China; 5 College of Foreign Languages, Guizhou Normal University, Guiyang 550001, China; cindymoon829@126.com; 6 Guizhou Provincial Center for Ecological and Environmental Monitoring, Guiyang 550081, China

**Keywords:** phytoplankton, functional groups, spatial heterogeneity, Nanming River

## Abstract

Rapid urbanization and industrialization have profoundly affected aquatic ecosystems in urban rivers, with phytoplankton taxa and functional group composition being particularly sensitive to environmental changes. Field surveys were conducted in the Nanming River, Guiyang, in October 2018 and July 2019, with 33 sampling sites evenly distributed across the upstream, midstream, and downstream reaches. The results revealed that: (1) The phytoplankton community comprised 6 phyla, 53 genera, and 61 species, dominated by Bacillariophyta, Chlorophyta, and Cyanobacteria. The community was classified into 20 functional groups, among which B, D, MP, P, and S1 were dominant and exhibited clear spatial heterogeneity along the longitudinal gradient. (2) Analysis of variance indicated that physicochemical parameters were the dominant factors explaining the variation in phytoplankton taxonomic and functional groups, with their independent contribution significantly higher than that of anthropogenic disturbance indicators and geographical factors. Redundancy analysis further identified NH_4_-N, TP, and TN as key environmental factors. Spearman’s correlation analysis further indicated that human activities alter ambient environmental conditions, which are significantly correlated with dissolved oxygen and chlorophyll a levels, thereby driving the differentiation of phytoplankton niches. (3) Functional group succession followed a distinct spatial pattern: upstream areas were dominated by groups P, SN, and Y, reflecting agricultural non-point source inputs; midstream areas were dominated by groups W1, H1, and S1, characteristic of urban complex pollution; and downstream areas were dominated by groups C and X1, indicating cumulative nutrient loading. Collectively, this study elucidates the driving mechanisms of phytoplankton dynamics in karst urban rivers and provides a scientific foundation for water quality monitoring, eutrophication risk pre-warning, and aquatic ecological restoration.

## Simple Summary

Phytoplankton are important primary producers in aquatic ecosystems, and understanding how their taxonomic and functional groups respond to multidimensional environmental drivers is crucial for assessing river health. This study reveals the spatial heterogeneity of phytoplankton taxonomic and functional groups in the Nanming River basin, with physicochemical parameters showing distinct upstream–downstream gradients. Our results indicate that physicochemical variables act as the primary drivers shaping the composition of phytoplankton taxonomic and functional groups, with stronger effects than anthropogenic disturbances and geographic features. Second, human activities alter aquatic environmental conditions and nutrient inputs, which further change chlorophyll a and dissolved oxygen via biological processes of primary producers, thereby affecting niche differentiation of phytoplankton taxonomic and functional groups. Therefore, watershed management for the Nanming River should prioritize nutrient load control and long-term monitoring of dominant diatom communities to track aquatic environmental variation. Overall, integrating regular physicochemical monitoring into urban river management helps predict algal community succession and mitigate potential eutrophication risks.

## 1. Introduction

Accelerating urbanization and industrialization are primary drivers of global riverine ecosystem degradation. These processes fundamentally restructure the physicochemical characteristics of aquatic environments through land-use changes and the influx of exogenous contaminants, leading to critical ecological consequences, including habitat fragmentation, nutrient enrichment, and heavy metal accumulation [1,2,3]. In the unique karst landscapes of Southwest China, fragile hydrogeological characteristics—including thin pedological veneers and dual-structured surface–groundwater interactions—exacerbate the vulnerability of river ecosystems to human interference. These features facilitate rapid nutrient leaching and contaminant transport, significantly increasing the complexity of ecological remediation [4,5].

Acting as both primary producers and biological “sentinels” for aquatic ecosystems, phytoplankton community dynamics and functional group structures exhibit rapid responses to nuanced variations in water physicochemical characteristics [6,7]. Phytoplankton succession not only serves as a direct indicator of water quality but also modulates energy flow and biogeochemical cycling across the entire ecosystem by regulating food web dynamics while remaining closely coupled with the risk of algal bloom outbreaks [8,9]. Consequently, elucidating how phytoplankton taxa and functional groups respond to multidimensional environmental drivers is pivotal to uncovering the mechanisms underlying ecological degradation in karst urban rivers and developing evidence-based management strategies.

The classification of phytoplankton functional groups, based on ecological strategies and resource utilization characteristics, provides a more accurate reflection of the adaptation between the community and the environment than traditional taxonomy [10]. Existing studies have confirmed that physicochemical factors, such as water temperature, nitrogen and phosphorus nutrients, and dissolved oxygen, are the core variables regulating phytoplankton community and functional group structures [11,12]. Meanwhile, trait-based research further reveals that phytoplankton functional group diversity is significantly lower under hyper-eutrophic conditions [13]. Similarly, taxonomic diversity also decreases under hypereutrophic conditions [14]. Nevertheless, previous studies are often limited to isolated environmental variables or non-karst river systems, resulting in a significant knowledge gap for karst urban watersheds. Specifically, in karst river systems, the synergistic interactions among geographical, physicochemical, and anthropogenic factors have not yet been systematically elucidated. Many studies are related with multi-factorial impact on river phytoplankton communities. For example, Rao et al. found that both nutrient-hydrological interactions and nutrient-temperature interactions significantly influence phytoplankton biomass and abundance [15]. Ding et al. found that water quality parameters (such as sediment and nutrient loads) are the primary drivers of changes in phytoplankton diversity [16]. Furthermore, the succession regularities of phytoplankton functional groups, along with the relative importance of their driving factors and their potential as ecological indicators, have yet to be fully elucidated [17,18]. In addition, the spatial mosaic of land-use types across the karst watershed—characterized by upstream agriculture, midstream urbanization, and downstream forestry—creates a distinct environmental pressure gradient. While this offers an ideal natural laboratory for investigating the spatial patterns of phytoplankton functional groups, focused research in this context is still lacking [19].

The Nanming River in Guiyang, a quintessential karst urban river, traverses a distinct landscape gradient encompassing agricultural catchments, densely populated urban cores, and forested reaches. These watershed-scale environmental gradients, coupled with the responsive dynamics of phytoplankton functional groups, epitomize the intricate ecological dynamics of karst river systems. Therefore, this study conducted systematic sampling and multidimensional analysis of the Nanming River at the watershed scale and proposed the following hypotheses: (1) the composition of phytoplankton taxonomic and functional groups exhibits significant spatial variation along the river’s longitudinal gradient (upper, middle, and lower); (2) physicochemical factors account for a larger proportion of community variation than geographical or anthropogenic variables. The findings of this study provide novel insights into the ecological evolutionary mechanisms of karst urban river ecosystems. Furthermore, they furnish a critical scientific basis and practical guidance for water quality assessment, eutrophication risk forecasting, and watershed-scale ecological remediation.

## 2. Materials and Methods

### 2.1. Study Area

The Nanming River, a major right-bank tributary of the Wujiang River, is affectionately hailed as Guiyang’s “Mother River.” The watershed spans a drainage area of 704.87 km^2^ (26°23′–26°45′ N, 106°32′–106°52′ E), encompassing Guiyang’s Huaxi, Nanming, Yunyan, and Wudang districts. Characterized by a rugged mountainous and hilly terrain with extensively developed karst formations—accounting for 82.28% of the total area—the landscape slopes gently from west to east. Land-use patterns exhibit a distinct spatial gradient: the upstream reaches are dominated by agricultural lands, the midstream constitutes a highly urbanized core under intense anthropogenic disturbance, and the downstream remains predominantly forested.

**Figure 1:**
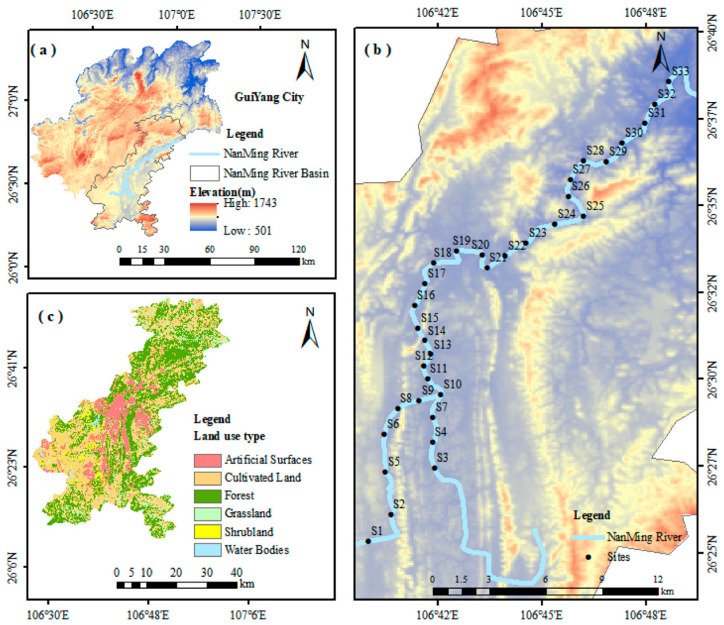
(**a**) Elevation of Guiyang City and the Nanming River Basin; (**b**) Land-use types of the Nanming River Basin; (**c**) Distribution of sampling sites along the Nanming River.

To characterize the spatial distribution of phytoplankton, field investigations were conducted in October 2018 and July 2019. Thirty-three representative sampling stations were established along the river’s main course (Figure 1), evenly distributed across three longitudinal segments: upstream (S1–S11), midstream (S12–S22), and downstream (S23–S33). This sampling scheme yielded a total of 66 samples across the study period (S1–S33 are sampling sites shown in Figure 1).

### 2.2. Sample Collection and Analysis

Phytoplankton samples (1.5 L) were collected at each site and immediately preserved with Lugol’s solution. Upon arrival at the laboratory, the samples were allowed to settle for 24–48 h, after which the supernatant was siphoned off to concentrate the samples to a final volume of ~30 mL [20]. For identification and enumeration, the concentrated samples were thoroughly homogenized, and a 100 μL aliquot was transferred to a 0.1 mL counting chamber. Phytoplankton cells were identified and counted across 100 randomly selected fields of view (FOV) under 400× magnification using a biological microscope (Olympus CX43, Shanghai, China) [21,22]. Taxonomic classification followed the protocols outlined in The *Freshwater Algae of China: Systematics, Taxonomy, and Ecology* [23]. In addition, a subset of samples was selected and treated with a mixture of concentrated nitric acid and concentrated sulfuric acid in a 1:1 volume ratio to prepare diatom slides. Each species was subsequently assigned to a functional group (FG) based on classification criteria established in previous studies [24,25].

Physicochemical parameters, including water temperature (WT), pH, conductivity (COND), and dissolved oxygen (DO), were measured in situ using a portable multi-parameter water quality analyzer (HANNA HI98194, Shenzhen, China). Nutrient concentrations—total phosphorus (TP), total nitrogen (TN), ammonium nitrogen (NH_4_-N), permanganate index (CODMn), chlorophyll a (Chl-a), and nitrate nitrogen (NO_3_-N)—were determined in accordance with the protocols specified in the *Environmental Quality Standards for Surface Water* (GB 3838-2002) [26]. To quantify anthropogenic disturbance intensity, socioeconomic variables—specifically Gross Domestic Product (GDP), Nighttime Light (NL), and land use types (artificial surfaces, cropland, forest, grassland, shrubland, and water bodies)—were incorporated into the analysis [27]. Spatially, a 30 m resolution Digital Elevation Model (DEM) was obtained from the Geospatial Data Cloud (https://www.gscloud.cn/ (accessed on 19 April 2025)). Using the Hydrology toolset in ArcGIS 10.8.1, sub-watersheds corresponding to each sampling site were delineated to compute the mean elevation. Source data for GDP, NL, and land use were retrieved from the Resource and Environment Science and Data Center website (https://www.resdc.cn/ (accessed on 31 July 2025)) and extracted for each sampling site using ArcGIS.

### 2.3. Statistical Analysis

One-way analysis of variance (ANOVA) was performed using SPSS Statistics 27 to assess spatial differences in physicochemical parameters. The Shannon diversity index and species richness index were calculated using the ‘vegan’ package (v2.6-4) in R 4.3.1 [28]. To elucidate the effects of environmental variables on phytoplankton taxonomic and functional groups, redundancy analysis (RDA) was conducted. Multicollinearity among variables was assessed using the variance inflation factor (VIF < 10); and a permutation test (999 permutations) was used to evaluate the significance of the RDA axes; non-metric multidimensional scaling (NMDS) was simultaneously employed to visualize compositional differences among sampling sections. Linear Discriminant Analysis Effect Size (LEfSe) was employed to identify discriminant taxa (biomarkers) among groups, with a logarithmic LDA score threshold of >2.5 and a significance level of α < 0.05. In addition, the ‘corr.test’ function in the ‘psych’ package of R was used to calculate Spearman’s rank correlation coefficients between the abundance of each phytoplankton taxonomic/functional group and each environmental factor. The resulting correlation matrix was visualized as a bubble plot (where bubble size is scaled by |r| and color indicates the sign and magnitude of r), retaining only statistically significant correlations (*p* < 0.05). Additionally, use the ‘psych’ package to analyze the relationships between dominant species and anthropogenic activities and physicochemical parameters.

The relative contributions of physicochemical, anthropogenic, and geographical factors to phytoplankton community variation were elucidated and visualized through variation partitioning analysis (VPA) combined with UpSet matrix plots. Phytoplankton biovolume is calculated according to cell volume and density [29].

(1) $$Biovolume=density\times volume\times abundance\times 10^{-9}$$

where the density of phytoplankton is 1 g/cm^3^; the unit of volume is um^3^; the unit of abundance is cells/L; and the unit of biomass is mg/L.

The McNaughton dominance index (*Y*) was used to identify dominant phytoplankton species, calculated as follows [30]:

(2) $$Y=\left(\frac{Ni}{N}\right)\cdot fi$$

where Ni is the number of individuals of species i; N is the total abundance of all phytoplankton species, and fi  is the occurrence frequency of species i  across all sampling sites. Phytoplankton species with Y≥0.02 were defined as dominant species, while those with Y≥0.1 were classified as absolute dominant species.

## 3. Results

### 3.1. Characteristics of Multidimensional Environmental Factors

**Figure 2:**
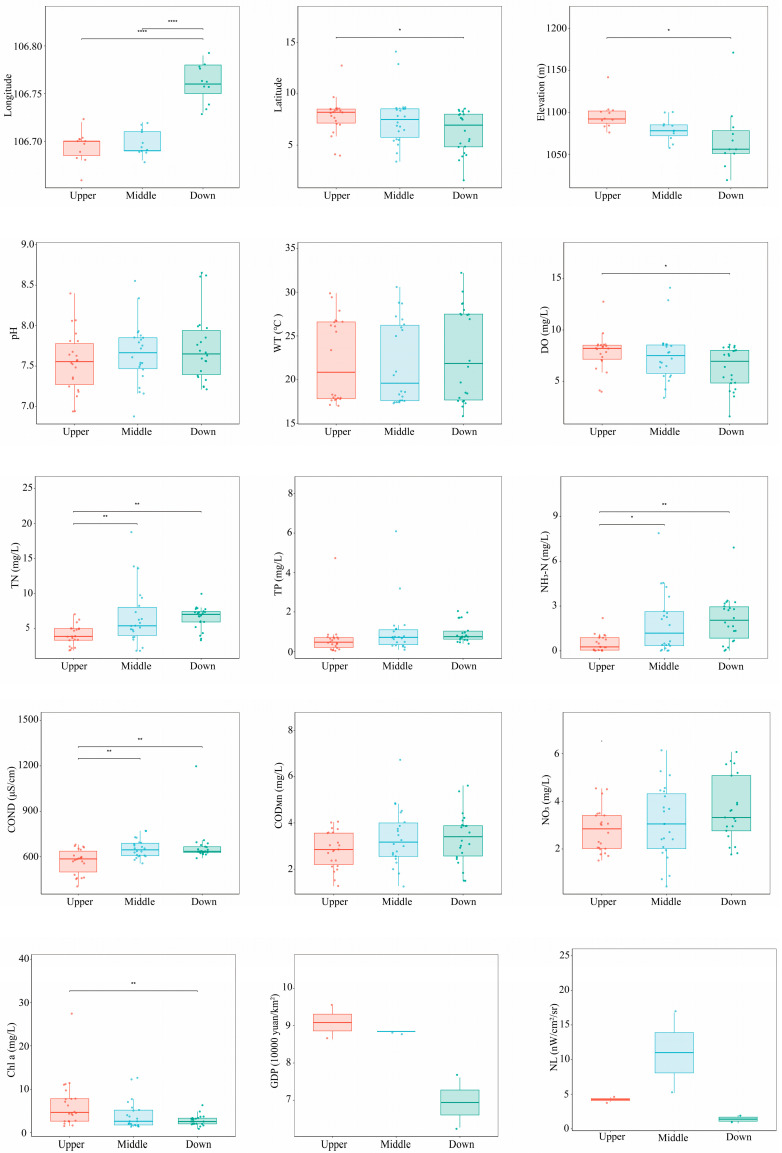
Distribution of geographical, physicochemical, and anthropogenic variables across the three sampling groups. (See the Materials and Methods Section for abbreviations. * indicates *p* < 0.05, ** indicates *p* < 0.01, **** indicates *p* < 0.0001).

Physicochemical properties and anthropogenic disturbance intensity were characterized for all sampling sites (Figure 1 and Figure 2). Geographically, both latitude and elevation decreased progressively from upstream to downstream. For physicochemical parameters, DO and Chl a concentrations were higher in the upper (DO: 7.82 ± 1.80 mg/L, Chl a: 6.61 ± 5.75 mg/L) and middle (DO: 7.38 ± 2.92 mg/L, Chl a: 4.10 ± 3.05 mg/L) than in the lower (DO: 6.30 ± 2.33 mg/L, Chl a: 2.95 ± 1.34 mg/L). Conversely, TN, COND, and NH_4_-N were significantly higher in the middle (TN: 7.05 ± 4.95 mg/L, COND: 642.6 ± 76.2 μs/cm, NH_4_-N: 2.39 ± 2.76 mg/L) and down (TN: 6.57 ± 1.84 mg/L, COND: 679.8 ± 117.5 μs/cm, NH_4_-N: 2.20 ± 2.12 mg/L) than in the upper (TN: 4.20 ± 1.80 mg/L, COND: 580.9 ± 80.8 μs/cm, NH_4_-N: 0.52 ± 0.60 mg/L). No significant spatial variations were detected in TP, WT, CODMn, pH, or NO_3_-N among the three sections.

Regarding anthropogenic activity indices, GDP and NL were higher in the upper (GDP: 9.08 ± 0.64 × 10^4^ yuan/km^2^, NL: 4.23 ± 0.45 nW/cm^2^/sr) and middle (GDP: 8.84 ± 0.10 × 10^4^ yuan/km^2^, NL: 10.9 ± 8.22 nW/cm^2^/sr) than in the lower reaches (GDP: 6.94 ± 0.95 × 10^4^ yuan/km^2^, NL: 1.40 ± 0.83 nW/cm^2^/sr). Land use composition exhibited distinct spatial heterogeneity: in the upper, the proportions of artificial surfaces, cropland, forest, grassland, shrubland, and water bodies were 9.43%, 43.1%, 32.7%, 9.30%, 4.60%, and 0.86%, respectively; in the middle, these proportions were 34.5%, 22.9%, 30.5%, 6.40%, 1.90%, and 0.46%, respectively; while the lower was dominated by forest (48.9%), followed by cropland (35.5%), grassland (11.9%), artificial surfaces (3.45%), water bodies (0.20%), and shrubland (0.05%). The lower anthropogenic indices downstream reflect a gradual reduction in human activity intensity along the lower reaches of this urban river.

### 3.2. Composition and Diversity of Phytoplankton Taxonomic Groups and Functional Groups

**Figure 3:**
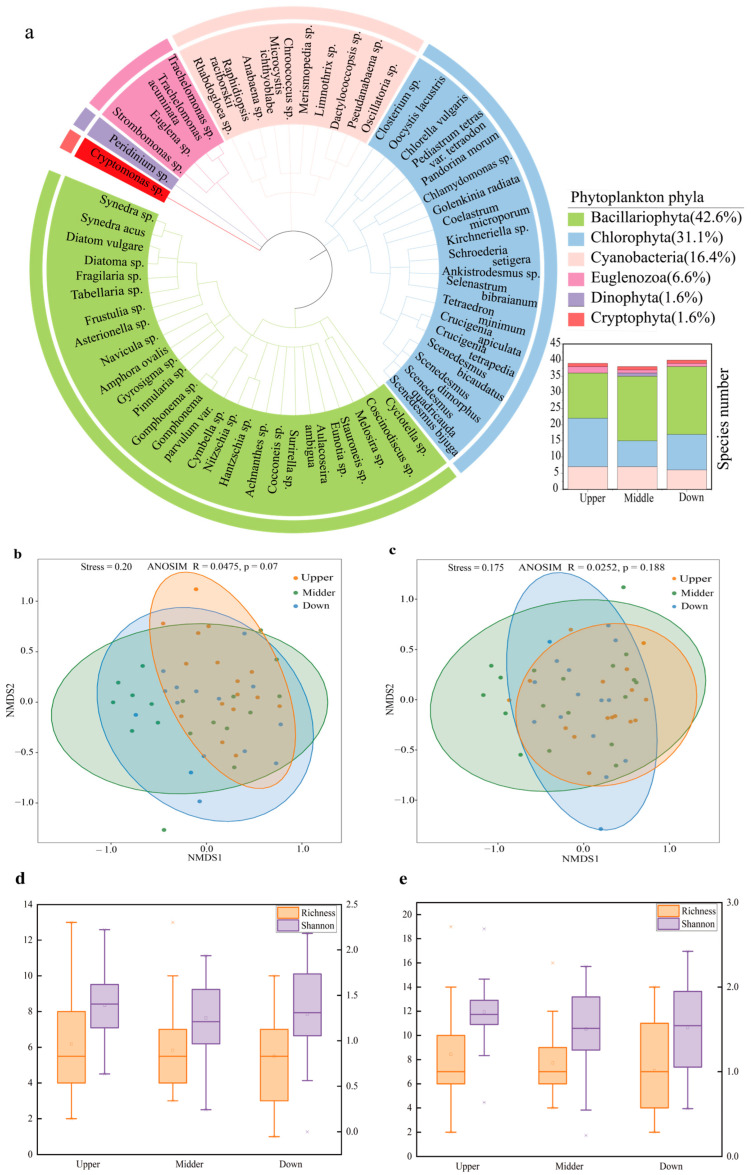
(**a**) Taxonomic tree of phytoplankton in the Nanming River. Non-metric Multidimensional Scaling (NMDS) ordination plots showing the compositional differences in phytoplankton taxa (**b**) and functional groups (**c**) across the three river sections. Bar charts displaying the alpha diversity indices of phytoplankton taxa (**d**) and functional groups (**e**).

**Figure 4:**
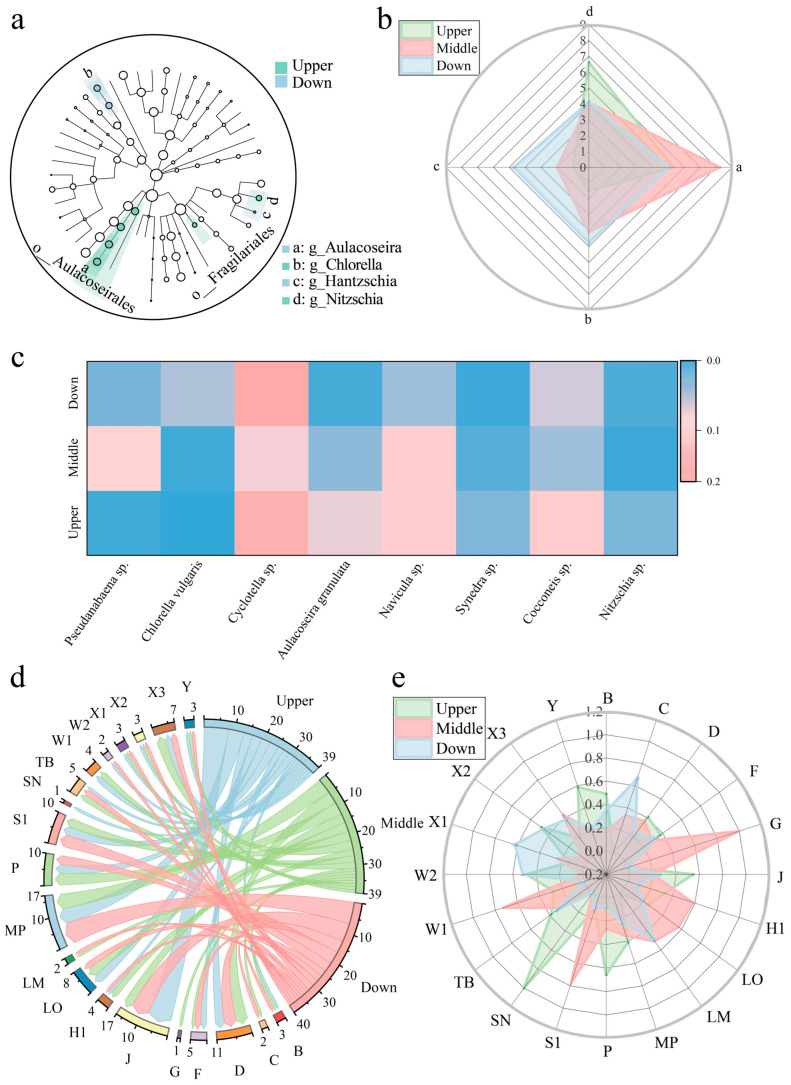
(**a**) LEfSe cladogram identifying discriminative phytoplankton taxa among the three river sections. (**b**) Niche breadth of the biomarkers identified by LEfSe for each section. (**c**) Distribution of dominant phytoplankton species across the three river sections. (**d**) Composition of phytoplankton functional groups. (**e**) Niche breadth of functional groups across the three river sections.

A total of 6 phytoplankton phyla were identified in this study: Chlorophyta, Bacillariophyta, Cyanobacteria, Dinophyta, Euglenophyta, and Cryptophyta. Phylum-level species composition analysis revealed that Bacillariophyta had the highest species richness (26 species, 42.6%), followed by Chlorophyta (19 species, 31.1%) and Cyanobacteria (10 species, 16.4%), while the remaining phyla accounted for relatively low proportions (Figure 3a). The community was dominated by three genera: Cyclotella, Navicula, and Cocconeis (Figure 4c). The occurrence of Navicula and Cocconeis, which are benthic diatoms, in the plankton samples may be attributed to scouring of the benthic substrate by water flow.

All identified taxa were classified into 20 functional groups (FGs) (Table 1) based on the phytoplankton functional group classification systems proposed by Reynolds et al. [31] and Padisák et al. [25], combined with their ecological traits and resource utilization strategies. In this study, FGs with a relative biomass contribution > 5.00% were designated as dominant. The results showed that FGs B (relative biomass: 0.17), D (0.09), MP (0.20), P (0.08), and S1 (0.28) were the core dominant groups. The heterogeneous distribution of these dominant FGs reflected the specialized ecological strategies of different taxa for adapting to heterogeneous environmental conditions.

Non-metric multidimensional scaling (NMDS) and ANOSIM were used to analyze the composition of taxonomic and functional groups in three river sections (upper, middle, and lower reaches). The NMDS stress value was 0.20 for taxonomic groups (Figure 3b) and 0.175 for functional groups (Figure 3c). The ANOSIM test (taxonomic groups: R = 0.0475, *p* = 0.07; functional groups: R = 0.0252, *p* = 0.188) revealed only minor spatial differences in phytoplankton community structure among the three river sections. Analysis of alpha diversity revealed a decreasing spatial gradient in species richness among phytoplankton taxa: upstream (6.18 ± 2.52) > midstream (5.82 ± 2.38) > downstream (5.50 ± 2.61); the Shannon diversity index was significantly higher in the upstream (1.39 ± 0.38) than in the midstream (1.25 ± 0.46) and downstream (1.29 ± 0.54) (Figure 3d). Consistent with the taxa-level patterns, functional group-level alpha diversity also showed an upstream-dominant pattern: both functional group richness (8.45 ± 3.81) and Shannon index (1.71 ± 0.42) in the upstream were higher than those in the midstream (7.73 ± 3.04, 1.50 ± 0.50) and downstream (7.09 ± 3.69, 1.52 ± 0.54), indicating greater functional diversity in the upstream reaches (Figure 3e).

### 3.3. Spatial Heterogeneity of Phytoplankton Taxonomic and Functional Groups

Along the upstream-to-downstream spatial gradient, the species compositions of Bacillariophyta, Chlorophyta, Cryptophyta, and Cyanobacteria showed distinct sectional variations (Figure 3a). To further elucidate the spatial heterogeneity of phytoplankton taxa, the Linear Discriminant Analysis Effect (LEfSe) was used to identify taxa with significant differences among the three river sections (Figure 4a). In the upstream section, the genera *Chlorella* and *Nitzschia* were identified as differentially abundant taxa. No taxonomic groups with significant differences in abundance were detected in the middle reaches of the river; this may be due to high intra-group heterogeneity or insufficient statistical power. In the downstream section, *Aulacoseira* and *Hantzschia* were identified as differentially abundant taxa. Niche breadth analysis of these taxa (Figure 4b) showed that *Nitzschia* exhibited the widest niche breadth (6.62) in the upstream section, followed by *Chlorella* (1.48). This calculation was performed at the genus level to reflect the overall environmental adaptation of the entire genus community rather than individual species within the genus. In the downstream section, the niche breadths of *Aulacoseira* and *Hantzschia* were 5.02 and 4.71, respectively. In addition, we found that the distribution of eight dominant phytoplankton taxa (dominance > 0.02) differed across the three river sections of the Nanming River (Figure 4c). Specifically, the total abundance was 14.5 × 10^6^ cells/L in the upper reaches, increased to 23.21 × 10^6^ cells/L in the middle reaches, and decreased to 10.21 × 10^6^ cells/L in the lower reaches, indicating that phytoplankton abundance varies with changes in the physicochemical environment (Figure 4c). Functional clusters also exhibit distinct characteristics in the upstream, midstream, and downstream regions (Figure 4d). Functional groups J and MP were relatively abundant in all three river sections, and niche breadth analysis (Figure 4e) showed that functional groups P, SN, and Y dominated the upstream section, with niche breadths of 0.66, 1.0, and 0.59, respectively; functional groups G, H1, W1, and S1 dominated the middle section, with niche breadths of 1.0, 0.58, 0.73, and 0.78, respectively; and functional groups C and X1 dominated the lower section, with niche breadths of 0.67 and 0.62, respectively. These results indicate that niche differentiation among functional groups is relatively pronounced in urban rivers under different physicochemical conditions. Notably, although phytoplankton samples were collected across summer and autumn, temporal effects were not statistically tested in the present spatial pattern analysis. This unaccounted seasonal variation may interfere with the interpretation of spatial heterogeneity, which represents an inherent limitation of this study.

### 3.4. Driving Factors of Phytoplankton Taxa and Functional Groups

**Figure 5:**
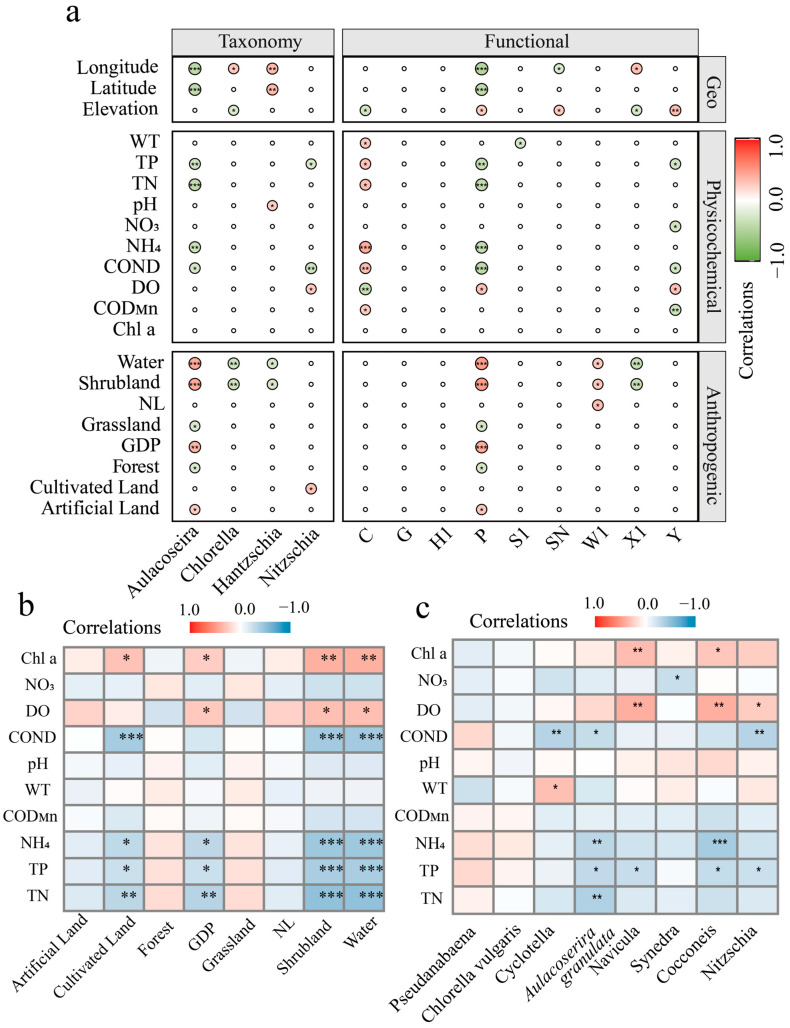
(**a**) Heatmap showing Spearman correlations between multiple influencing variables and discriminative species/functional groups in the Nanming River. (**b**) Relationships between anthropogenic activity indices and physicochemical parameters. (**c**) Relationships between the abundance of dominant species and physicochemical factors. * indicates *p* < 0.05, ** indicates *p* < 0.01, *** indicates *p* < 0.001.

This study further employed Spearman’s correlation analysis to reveal the relationships between the distinct taxonomic groups and functional groups in the upper, middle, and lower river sections and various environmental factors (Figure 5). Overall, geospatial factors, physicochemical parameters, and anthropogenic disturbance indices all exerted significant impacts on phytoplankton distribution patterns. At the taxonomic level, *Aulacoseira* was significantly positively correlated with artificial surfaces, GDP, shrubland and water bodies; such positive association is largely attributable to eutrophication-induced excessive proliferation of *Aulacoseira* under anthropogenic land-use disturbance. *Hantzschia* was positively correlated with pH, latitude, and longitude; and *Nitzschia* exhibited significant positive correlations with cropland and DO. At the functional group level: FG C was positively correlated with COD_Mn_, COND, NH_4_-N, WT, TP, and TN; FG P showed significant positive correlations with artificial surfaces, GDP, shrubland, water bodies, DO, and elevation; FG W1 was positively correlated with night-time light (NL), shrubland, and water bodies (Figure 5a). Meanwhile, correlations between anthropogenic factors and physicochemical parameters revealed that cropland, GDP, shrubland, and water bodies were significantly negatively correlated with TN, TP, NH_4_-N, and COND, but positively correlated with DO and Chl-a (Figure 5b). Furthermore, correlation analysis between the abundance of dominant species and physicochemical parameters revealed that *Navicula* sp., *Cocconeis* sp., and *Nitzschia* sp. showed significant positive correlations with chlorophyll a and dissolved oxygen, which is reasonable given that these taxa constitute the dominant primary producers and contribute largely to aquatic algal biomass; meanwhile, *Cyclotella* sp. was significantly positively correlated with water temperature (WT) (Figure 5c). These results further confirm that nutrient enrichment is the primary driver of community biomass variation in this watershed, implying the potential impacts of human activities on the physicochemical conditions of urban rivers.

**Figure 6:**
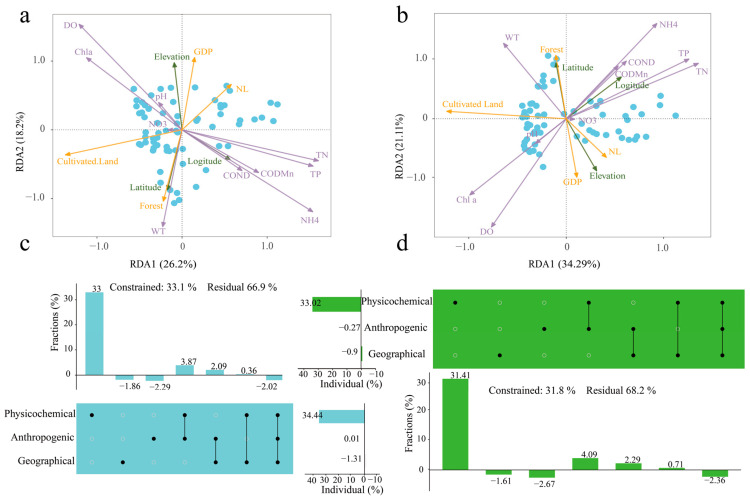
Redundancy Analysis (RDA) illustrating the effects of multiple factors on phytoplankton Taxa (**a**) and functional groups (**b**). UpSet matrix plots were employed to visualize the results of variance partitioning, illustrating the unique contributions of geographical, physicochemical, and anthropogenic factors to the variance in phytoplankton taxa (**c**) and functional groups (**d**).

Redundancy Analysis (RDA) was used to further quantify the contributions of influencing factors to phytoplankton taxa and functional groups in the Nanming River (Figure 6). Based on the vector characteristics of environmental factors, nutrient concentrations (TN, TP, NH_4_-N) exhibited strong positive correlations with organic pollution indices (COD_Mn_, COND), indicating that nutrient enrichment and organic pollution are the core environmental gradients driving phytoplankton taxa (Figure 6a). Similarly, NH_4_-N, TP, TN, and COD_Mn_ showed strong positive correlations with functional groups, while land use patterns and geographical factors also demonstrated significant explanatory capacity. Notably, GDP and NL (representing urbanization levels) were aligned in the same direction as elevation, suggesting that the coupling of high-intensity anthropogenic disturbance with specific topographical conditions may exert potential pressure on the aquatic ecosystem (Figure 6b). In summary, the spatial heterogeneity of phytoplankton functional groups in the Nanming River is primarily governed by two key gradients: a water quality gradient dominated by nitrogen and phosphorus nutrients and a complex anthropogenic disturbance gradient composed of urbanization and agricultural activities.

The results of the variance decomposition indicate that environmental factors collectively account for 33.1% of the variation in phytoplankton community structure, while the remaining 66.9% of the variation remains unexplained. This suggests that unquantified biotic and abiotic factors (such as seasonal changes) may have played a significant role in community assembly. It should be noted that the sample in this study covers only the summer and fall seasons and does not include spring and winter; this is one of the limitations of this study. In terms of the independent contributions of each factor, physicochemical factors accounted for 33% of the variation in phytoplankton taxonomic groups, while anthropogenic and geographic factors contributed −2.29% and −1.86%, respectively (Figure 6c). These negative values are likely due to multicollinearity among the factor groups. Similarly, variations in functional groups were also primarily explained by physicochemical parameters (31.41%), with relatively smaller contributions from anthropogenic and geographic factors (Figure 6d). Taken together, these results indicate that physicochemical parameters are the primary factors regulating the variation in phytoplankton taxonomic and functional groups in this urban karst river.

## 4. Discussion

### 4.1. Analysis of Spatial Heterogeneity of Taxa and Functional Groups

The phytoplankton taxa and functional groups in the Nanming River displayed significant spatial heterogeneity along the upstream-to-downstream gradient. Regarding species composition, a clear successional trajectory was observed across the entire watershed: a shift from mixed diatom–chlorophyte dominance in the upstream reaches to diatom predominance in the midstream and downstream [32]. Notably, *Nitzschia* exhibited an exceptionally broad niche breadth and a significantly positive correlation with dissolved oxygen (DO). This is consistent with findings from the Yellow River Basin [33], suggesting that despite heterogeneous nutrient inputs induced by urbanization upstream, the high self-purification capacity (indicated by high DO levels) sustained specific community characteristics. In the midstream urban core area where anthropogenic disturbance was the most intense, the phytoplankton community showed a distinct absence of characteristic indicator taxa. This phenomenon reflects the complexity of environmental pressures in the midstream, where only phytoplankton species with strong adaptability and broad tolerance could survive [33,34]. In the downstream reaches, the characteristic taxa shifted to *Aulacoseira* and *Hantzschia*; correlation analysis indicated that these taxa were regulated by the combined effects of local anthropogenic activities (e.g., GDP, land use), basin-wide material transport, and natural recovery capacities [35,36].

Niche analysis further revealed that the niche breadths of upstream *Nitzschia* and downstream *Hantzschia* were both wider than those of non-biomarker taxa, whereas *Aulacoseira* in the downstream reaches showed the opposite niche pattern. This discrepancy indicates a marked divergence in the relative roles of local environmental heterogeneity and species intrinsic traits in mediating community assembly across different river segments [37]. Heterogeneous fluvial environments further stimulated niche differentiation among taxa within each segment [38]. Specifically, this study found that the abundance of the aforementioned benthic diatoms (*Cyclotella*, *Navicula*, *Cocconeis*, and *Nitzschia*) was significantly positively correlated with dissolved oxygen, chlorophyll a, and water temperature (Figure 6c). It should be noted that these diatoms are not typical planktonic bloom species; their relatively high abundance in the water column is primarily due to their massive growth on attached substrates, followed by their being washed into the water column by water currents. Given that nutritional indicators (such as chlorophyll a) are primarily driven by anthropogenic factors, including land cover and regional GDP levels, future watershed management must prioritize the simultaneous monitoring of anthropogenic disturbance sources and key species responsible for algal blooms—an approach that is critical to maintaining the sustainability of urban aquatic ecosystems.

Phytoplankton aggregation was notably denser in the downstream regions (Figure 2), and this “high abundance, low diversity” pattern (Figure 3) arose from the combined effects of nutrient accumulation, anthropogenic disturbance, and site-specific hydrological conditions [39,40,41]. Specifically, under eutrophic conditions, hydrological characteristics—particularly low flow velocity—played a pivotal role: the extended hydraulic retention time provided sufficient reproductive cycles for slow-growing species such as cyanobacteria [42]. Therefore, co-monitoring anthropogenic variables (e.g., land use, GDP) and key bloom-forming species represents a core strategy for mitigating downstream bloom risks and safeguarding aquatic ecological security.

Phytoplankton functional groups (FGs) exhibited distinct spatial responses to heterogeneous environmental pressures. In the upstream reaches, FG P showed a positive correlation with artificial land cover, nighttime light (NL), and shrubland, indicating that nutrient inputs associated with rural urbanization have initiated alterations to the relatively undisturbed aquatic community structure. Endowed with high light use efficiency and resistance to physical disturbance, FG P occupied a dominant niche in habitats with high DO levels, rapid flow velocities, and increasing nutrient enrichment [43,44]. In the midstream—a region of intensive urbanization where NL acts as a direct proxy for anthropogenic activity—FG W1 (characterized by a mixotrophic life history) dominated the phytoplankton community under the influence of habitat fragmentation. This functional group efficiently utilizes organic carbon sources from urban sewage and adapts to light-limited environments induced by bridge shading and high turbidity, and its dominance highlights the filtering effect of urban landscapes on species with high functional plasticity [45].

A striking feature of the downstream reaches was a highly significant positive correlation between FG C and COD_Mn_, NH_4_-N, TN, TP, and electrical conductivity (COND). This demonstrates that nutrient accumulation becomes the primary driver of phytoplankton community variation as the river flows downstream. The downstream section receives artificial runoff and domestic sewage from the upstream and midstream, forming a typical habitat characterized by high nutrient loads; FG C, which is highly sensitive to nitrogen and phosphorus fluctuations, proliferates rapidly under conditions of suitable WT and low flow velocity, thereby becoming the core characteristic of the downstream phytoplankton community [46]. In summary, the spatial succession of phytoplankton functional groups in the Nanming River not only objectively reflects the environmental gradient changes along the river but also provides critical theoretical support at the functional level for river health management and the implementation of targeted ecological regulation strategies.

### 4.2. Driving Factors of Variation in Phytoplankton Taxa and Functional Groups

Physicochemical factors, hydrological characteristics, climatic variability, and anthropogenic activities collectively shape phytoplankton community patterns in urban rivers [47]. In this study, by visualizing variance partitioning analysis (VPA) results via UpSet plots, we identified physicochemical parameters as the core drivers of variation in phytoplankton taxa and functional groups in the Nanming River. Their independent explanatory power was significantly greater than that of anthropogenic activities and geographical factors, a finding consistent with recent investigations of typical urbanized rivers [48]. This suggests that at the scale of urbanized rivers, phytoplankton community variation mainly arises from the interaction between dispersal limitation and regional habitat heterogeneity [49]. Specifically, dispersal limitation highlights that geographical distance and anthropogenically introduced physical barriers (e.g., rubber dams and landscape impoundments) elevate resistance to species migration, resulting in spatial differentiation of phytoplankton communities among river segments. In contrast, regional habitat heterogeneity is manifested as pronounced physicochemical gradients (notably nutrient concentrations and WT) that exert strong environmental filtering effects, thus governing the deterministic processes of phytoplankton community assembly [50].

Second, nutrients (NH_4_-N, TP, TN) were further identified as key factors mediating variations in phytoplankton taxa and functional groups, a finding consistent with the general consensus in urban river ecology [51,52]. Urban wastewater and surface runoff introduce significant amounts of nutrients, alleviating growth limitations associated with oligotrophic conditions, laying the foundation for changes in phytoplankton communities, and leading to an increase in pollution-tolerant dominant species [53]. This shift in nutrient gradients directly mediated the successional transition of phytoplankton functional groups from FG P (upstream) to FG C (downstream). FG C’s high sensitivity to nutrient fluctuations allows it to rapidly establish absolute dominance in downstream high-nutrient habitats, a process governed by the “trait filtering” mechanism [54].

Furthermore, Spearman’s rank correlation analysis revealed a positive correlation between forest land cover and several nutrient indices. This result challenges the traditional “forest nutrient retention” paradigm [55] and instead profoundly reflects the unique characteristics of the karst geological background. Karst regions are characterized by shallow soil layers and a dual surface–subsurface hydrological structure; agricultural non-point source pollution and domestic sewage readily infiltrate the groundwater system via vertical conduits (e.g., dissolution fissures and sinkholes). Ultimately, these pollutants discharge into rivers via springs or baseflow [56], resulting in elevated background nutrient concentrations even in forested areas. Thus, it is clear that in karst urbanized rivers, phytoplankton community assembly is not governed by single geospatial scale factors but rather by a coupled driving mechanism involving local nutrient inputs (i.e., physicochemical factors) and the unique hydrogeological setting of karst regions.

### 4.3. Ecological Implications

Phytoplankton, as key regulators of ecological balance and nutrient cycling in fluvial ecosystems [57], play a pivotal role in sustaining aquatic ecosystem health. Shifts in their community composition and functional traits act as sensitive bioindicators, providing profound insights into the response mechanisms of urban rivers to anthropogenic environmental pressures. However, intensified urbanization has increased exogenous nutrient inputs and elevated concentrations of NH_4_-N and phosphorus, which in turn alters the competitive dynamics of phytoplankton communities, facilitates the dominance of pollution-tolerant taxa (e.g., certain cyanobacterial species), and elevates the risk of algal blooms [58,59].

Accordingly, future river management strategies must be grounded in a comprehensive understanding of phytoplankton taxa and functional characteristics. It is therefore essential to integrate anthropogenically driven variations in physicochemical factors into algal bloom risk prediction frameworks. Simultaneously, optimizing land use management at the watershed scale—via precise control of nutrient fluxes and the implementation of targeted water quality improvement measures—is critical to safeguarding the sustainability of ecological functions and services in urban fluvial ecosystems.

### 4.4. Limitations and Future Perspectives

This study has several notable limitations. First, samples were only collected in summer and autumn, with no specimens obtained in spring and winter. Although our dataset covers two different seasons, we did not conduct statistical analyses to distinguish and quantify independent temporal effects when interpreting spatial distribution patterns. The limited seasonal sampling coverage prevents a full characterization of the annual seasonal succession patterns of phytoplankton.

Second, algal identification was solely based on microscopic morphological traits without the assistance of molecular biological methods for taxonomic validation. Specifically, due to the high morphological similarity of closely related species and practical experimental restrictions, most diatom taxa were only identified to the genus instead of species level. This is a common drawback of routine field ecological surveys of rivers.

For future research, year-round continuous monitoring and sampling across four seasons are recommended. Combined application of molecular sequencing techniques can improve the accuracy of algal taxonomic identification, thereby advancing research on phytoplankton communities in karst urban rivers.

## 5. Conclusions

This study elucidates the response patterns of phytoplankton taxa and functional groups to varying physicochemical conditions in urban rivers. The results show that phytoplankton in the Nanming River can be classified into 20 functional groups, with the dominant groups being B, D, MP, P, and S1, which exhibit distinct spatial heterogeneity along the longitudinal gradient of the river. Notably, physicochemical factors exert a stronger influence on phytoplankton taxa and functional group succession compared to geographic and anthropogenic factors. In addition, human activities have influenced the niche differentiation of phytoplankton taxonomic and functional groups by altering physicochemical conditions. These findings provide insights into the management of the Nanming River ecosystem as an urban river.

## Figures and Tables

**Table 1:** Phytoplankton species and functional groups in the Nanming River. (* indicates dominant functional groups).

Functional Groups	Phytoplankton Species	Habitat Template
B *	*Cyclotella* sp.	Medium trophic, small-to-medium, or large shallow-water bodies
C	*Asterionella* sp.	Eutrophic small- and medium-sized lakes with species sensitive to the onset of stratification
D *	*Synedra* sp., *Synedra acus.*, *Hantzschia* sp., *Nitzschia* sp., *Coscinodiscus* sp.	Rich in nutrients, cloudy
F	*Kirchneriella* sp., *Oocystis* sp., *Selenastrum* sp.	Medium-to-rich in nutrients, clean, and strong water mixing
G	*Pandorina* sp.	Eutrophic, stagnant water bodies
J	*Scenedesmus* sp., *Crucigenia* sp., *Coelastrum* sp., *Tetraedron* sp., *Pediastrum* sp., *Golenkinia* sp.	Shallow, mixed, highly enriched systems
H1	*Anabaena* sp., *Diploneis ovalis.*	Eutrophic, both stratified and shallow lakes with low nitrogen content
LO	*Merismopedia* sp., *Chroococcus* sp. *Microcystis* sp., *Peridinium* sp.	Eutrophic to hypertrophic, small to medium-sized lakes
LM	*Dactylococcopsis* sp.	Deep and shallow, oligo to eutrophic, medium to large lakes
MP *	*Oscillatoria* sp., *Navicula* sp., *Achnanthes* sp. *Cocconeis* sp., *Surirella* sp., *Pinnularia* sp., *Stauroneis* sp., *Eunotia* sp., *Cymbella* sp.	Frequently stirred up, inorganically turbid shallow lakes
P *	*Melosirab* sp., *Fragilaria* sp., *Closterium* sp., *Diatoma* sp., *Tabellaria* sp.	Continuous or semi-continuous mixed bodies of water, higher trophic states
S1 *	*Pseudanabaena* sp., *Rhabdogloea* sp., *Limnothrix* sp., *Planktothrix* sp.	The mixture is cloudy and has low transparency
SN	*Cylindrospermopsis* sp.	Warm mixed environments
TB	*Gomphonema* sp., *Gomphonema parvulum var. lagenula*	Highly lotic environments (streams and rivulets).
W1	*Euglena* sp., *Euglena oxyuris*	Ponds, even temporary, rich in organic matter from husbandry or sewages
W2	*Trachelomonas* sp., *Strombomonas* sp.	Medium nutrition, shallow water
X1	*Chlorella* sp., *Ankistrodesmus* sp.	Shallow, eutrophic-hypertrophic environments
X2	*Chlamydomonas* sp.	Shallow, meso-eutrophic environments; sensitive to mixing, filter feeding; tolerant of stratification
X3	*Schroederia* sp., *Frustulia* sp., *Gyrosigma* sp.	Shallow, well mixed Oligotrophic environments
Y	*Cryptomonas* sp.	Still-water environment

## Data Availability

All data were included in the paper.
